# Endogenous mucosal phosphatases characterization in duodenum brush border membrane of laying hens

**DOI:** 10.3389/fphys.2025.1581088

**Published:** 2025-04-02

**Authors:** Anna Hanauska, Vera Sommerfeld, Margit Schollenberger, Korinna Huber, Markus Rodehutscord

**Affiliations:** Institute of Animal Science, University of Hohenheim, Stuttgart, Germany

**Keywords:** age, brush border membrane, genetic strain, *in vitro* assay, laying hen, mucosal phosphatase, phosphorus, phytate degradation

## Abstract

Chicken mucosal phosphatases can partially degrade phytate contained in the feed. Little is known about the characteristics and degradation products of such mucosal phosphatases and the effects of age and genetic strain of the chicken. The objective of this study was to characterize endogenous mucosal phosphatases of two laying hen strains fed diets with or without mineral phosphorus (P) before and after the onset of egg laying. Hens of the strains Lohmann Brown-classic (LB) and Lohmann LSL-classic (LSL) were sacrificed in weeks 19 and 24 of age after 4 weeks of feeding one of two diets with (P+) or without (P-) mineral P supplement. Mucosa of the duodenum was collected, and the brush border membrane (BBM) of enterocytes was enriched and used for phosphatase activity determination. Additionally, the BBM was used in a modified three-step *in vitro* assay to study the InsP_6_ degradation products. The results of both *in vitro* assays were not significantly affected by hen strain and diet. The activity of mucosal phosphatase in 19-week-old hens was, on average, 0.8 *µ*mol P_i_/g BBM protein/min lower than in 24-week-old hens (*P* < 0.002). Consistently, the InsP_6_ concentration in the incubation residue was significantly higher in 19-week-old hens than in 24-week-old hens (*P* < 0.001). In the incubation residue, the concentrations of Ins(1,2,3,4,5)P_5_, Ins(1,2,3,4,6)P_5_, and Ins(1,2,3,4)P_4_ were significantly lower (*P* ≤ 0.002), and those of InsP_3_ and InsP_2_ were significantly higher (*P* ≤ 0.027) when BBM of 24-week-old hens was used compared to 19-week-old hens. The InsP_6_ degradation products suggest the activity primarily of a 6- and secondarily of a 5-phytase in the duodenal mucosa. The consistent results from both *in vitro* assays provide a comprehensive characterization of these enzymes. Under the conditions of this study, small intestine calcium concentration appeared to influence mucosal enzyme activity more than dietary mineral P supplementation.

## 1 Introduction

Phytic acid (*myo*-inositol 1,2,3,4,5,6-hexakis (dihydrogen phosphate); InsP_6_) and its salt phytate is the main binding form of phosphorus (P) in plant feedstuffs (Eeckhout and De Paepe, 1994). For InsP_6_ hydrolysis in the digestive tract and in order to make the phytate-P available for the animal, phytate-degrading enzymes called phytases and other phosphatases are needed. Phytases may be contained in feed ingredients or added in feed formulation but endogenous phytases also exist, either produced by the epithelial cells or microorganisms in the digestive tract.

In poultry, the endogenous phytase and phosphatase activity has long been considered to be insufficient to degrade a marked amount of phytate (Nelson et al., 1968; Singh, 2008). A comprehensive body of information from more recent studies suggests a high biological potential for endogenous InsP_6_ degradation exists, especially in broiler chickens and turkeys (Rodehutscord et al., 2022). However, variation in the extent of endogenous InsP_6_ degradation among the studies was high (Rodehutscord et al., 2022). The concentrations of calcium (Ca) and P in the feed, varied alone or in combination, contributed to such variation (Tamim et al., 2004; Bedford and Rousseau, 2017; Sommerfeld et al., 2019; Novotny et al., 2023a). The extent of InsP_6_ degradation overall is much lower in laying hens with up to 47% (Scheideler and Sell, 1987; Van der Klis et al., 1997; Carlos and Edwards, 1998; Abudabos, 2012) than in broiler chickens with up to 90% (Rodehutscord et al., 2022), whereby the level of endogenous phosphatase activity is considered to be generally higher in laying hens than in broilers (Maenz and Classen, 1998). The biological meaning of these obviously contradictory observations is not clear yet, but lower InsP_6_ degradation might be caused by the higher Ca concentration of the laying hen diet (Van der Klis et al., 1997) leading to the formation of less soluble InsP_6_-Ca complexes (Angel et al., 2002). The age of laying hens influenced the endogenous phosphatase activity, but the effects were inconsistent and included both an increase and a decrease with increasing age (Marounek et al., 2008; Abudabos, 2012; Sommerfeld et al., 2020). Moreover, laying hens from different genetic strains exerted differences in endogenous phosphatase activity (Sommerfeld et al., 2020).

While some research has been conducted on the activity of endogenous mucosal phosphatases, the degradation pathways of the endogenous enzymes remain largely unexplored, and the quantitative contributions of microbial and mucosal phosphatases to endogenous InsP_6_ degradation have not been clarified (Rodehutscord et al., 2022). The reason for this lack of studies may be the experimental challenges in distinguishing microbial and mucosal enzymes. *In vivo* studies of the degradation cascade of mucosal phosphatases need the use of germ-free animals, which implies a high experimental effort. *In vitro* assays are an alternative to *in vivo* experiments for studying InsP_6_ degradation (Pontoppidan et al., 2012) under well-standardized conditions with the exclusion of gut microbiome effects (Sommerfeld and Santos, 2021).

The animal sample material used in this *in vitro* study was derived from the animal study conducted by Sommerfeld et al. (2024), which investigated the effect of mineral P on phytate degradation in two laying hen strains before and after the onset of laying activity. The *in vivo* study demonstrated that older laying hens displayed an increased body weight and feed intake, and Lohmann Brown-classic (LB) hens showed a higher body weight, feed intake and egg weight than Lohmann LSL-classic (LSL) hens. Interactions among the factors studied were observed for P concentrations in duodenum + jejunum and ileum digesta and P retention. We hypothesize that endogenous phosphatases play a predominant role in P utilization, aiming to enhance the understanding of differences between laying hen strains and with the long-term aim of reaching the optimal production efficiency whilst maintaining their best health.

The main objective of this study was to characterize endogenous mucosal phosphatases of laying hens, by determining the InsP_6_ degradation pattern and phosphatase activity. For this purpose, an enzyme activity assay was combined with a modified *in vitro* InsP_6_ degradation assay. The second objective was to compare two different genetic strains of laying hens at two ages when fed diets with or without mineral P supplementation. The first hypothesis was that the mucosal enzyme activity and hydrolysis products are affected by the strain and age of the laying hens. The second hypothesis was that adding mineral P to the feed reduces the *in vitro* degradation of InsP_6_.

## 2 Materials and methods

### 2.1 Animals, procedures, and sampling

This study was part of the interdisciplinary Research Unit P-Fowl–Inositol phosphates and *myo*-inositol in the domestic fowl: Exploring the interface of genetics, physiology, microbiome, and nutrition (https://p-fowl.uni-hohenheim.de/). The animal trial was performed in accordance with the German animal welfare legislation following approval by the Regierungspräsidium Tübingen, Germany (Project no. HOH67-21TE). Details of the trial concerning the design, animals involved, and diets fed have been described by Sommerfeld et al. (2024). In brief, the trial implied a 2 × 2 × 2-factorial arrangement of treatments. Hens of the strains LB (n = 40) and LSL (n = 40) were used. At the age of 15 and 20 weeks, hens from each strain were placed individually in metabolic units and 10 hens per strain were assigned one of two dietary treatments (without [P-] and with [P+] mineral P supplement) in a randomized complete block design. The P supplement was monocalcium phosphate (MCP) included at 1 g P/kg of diet. The analyzed P, Ca and non-phytate-P concentrations were 3.6, 38.2, and 1.8 g/kg DM for P-, and 4.9, 35.7, and 3.1 g/kg DM for P+ (Sommerfeld et al., 2024). The diets were corn and soybean meal-based and provided to the hens for *ad libitum* consumption. In weeks 19 and 24, the respective hens were stunned with a gas mixture (35% CO_2_, 35% N_2_, 30% O_2_) and killed by decapitation and exsanguination. The age was chosen to represent the periods shortly before and after the onset of laying activity to consider the effects of egg production on the mineral metabolism of hens.

Immediately after exsanguination, the duodenum was excised and digesta was removed by gentle pressing with the thumb and indicator. Then, the duodenum was cut open longitudinally, flushed in cold physiological saline (0.9% NaCl), and enrolled on a glass plate placed on ice. Mucosa from the duodenum section was stripped off from the muscle layer with microscopic slides and immediately shock-frozen in liquid nitrogen, transported in liquid nitrogen to the laboratory, and stored at −80 °C (HERAfreeze™ HFU T Series −86 °C Thermo Scientific™, Fisher Scientific GmbH, Schwerte, Germany) until further processing.

### 2.2 Brush border membrane preparation

The brush border membrane (BBM) of duodenal enterocytes was enriched as described by Huber et al. (2015) with slight modifications. First, the entire mucosa obtained from each hen was ground in liquid nitrogen with a mortar and pestle. The ground mucosa was weighed in 0.5 g portions into a glass potter vessel (Homogenisatorgefäß, neoLab Migge GmbH, Heidelberg, Germany), mixed with 4-(2-hydroxyethyl)piperazine-1-ethanesulfonic acid/mannitol buffer (HEPES 2 mM, mannitol 50 mM, PMSF 25 mM), and homogenized in a glass potter with the appropriate homogenizer (homogen^plus^, shuett-biotec GmbH, Göttingen, Germany). To separate the BBM, the basolateral membrane was precipitated by adding 1 M MgCl_2_ to the mucosal homogenates. The basolateral membrane precipitates were removed by centrifugation for 20 min at 3,000 × *g* (Sorvall LYNX 4000 Superspeed-Centrifuge, Thermo Scientific™, Life Technologies GmbH, Darmstadt, Germany) after shaking the samples on ice for 20 min at 80 rpm (MS Rocking Shaker, MS-NRK-30, Major Science Co., Ltd., Taiwan). The addition of 1 M MgCl_2_, followed by shaking and centrifugation, was conducted twice. Enriched BBM pellets were obtained with subsequent centrifugation at 30,000 × *g* for 30 min. The BBM pellets were resuspended in HEPES/mannitol buffer (HEPES 20 mM, mannitol 300 mM, MgSO_4_ 0.1 mM) including protease inhibitor (cOmplete™ Mini Protease Inhibitor Cocktail, Roche Diagnostics GmbH, Mannheim, Germany). Then BBM aliquots (50–140 *µ*L) were frozen and stored at −80 °C. The BBM protein was quantified in triplicate using the Bradford assay (Bradford Reagent, 5 x, SERVA, Heidelberg, Germany).

### 2.3 InsP_6_ degradation assay

A three-step *in vitro* assay described by Sommerfeld et al. (2017) was modified for the purpose of this study. The assay considers crop, stomach, and small intestine conditions by simulating temperature, pH, gastric and pancreatic enzymes, and mean retention times. Briefly, a mixture of corn and soybean meal was used as the substrate providing 11.0 *µ*mol InsP_6_/g, 0.6 *µ*mol Ins(1,2,4,5,6)P_5_/g, and 0.2 *µ*mol Ins(1,2,3,4,5)P_5_/g (Table 1). An amount of 0.1 g of the mixture was weighed into 10 mL tubes. In step 1, 150 *µ*L double-distilled water and 8.5 *µ*L of 0.25 M HCl were added to achieve a pH of 5.8 and sufficient moistening of the substrate. After vortexing and adding two glass beads (Glass beads 5 mm, Merck KGaA, Darmstadt, Germany) per tube, samples were incubated for 30 min at 40 °C in a shaking water bath with a 20 L water chamber (Shaking Water Baths GFL-1083, GFL Gesellschaft für Labortechnik mbH, Burgwedel, Germany). In step 2, 5 *µ*L double-distilled water containing 300 U pepsin (from porcine gastric mucosa, 77160-100G, Sigma-Aldrich, St. Louis, MO, United States) and 160 *µ*L 0.25 M HCl were applied to achieve a pH of 2.8 and, after vortexing, samples were incubated again for 45 min at conditions as in step 1. Step 3 started with the addition of 38.5 *µ*L pancreatin solution (3.7 mg pancreatin/mL 1 M NaHCO_3_ (from porcine pancreas, P7545-25G, Sigma-Aldrich, St Louis, MO, United States)) at pH 6.1. After vortexing, the BBM solution standardized at an amount equivalent to 1,600 *µ*g BBM protein was added, and the mix was then incubated for 60 min. This amount of BBM was identified in previous test runs of this assay and ensured that the amount of InsP_6_ from the substrate was not a limiting factor for the enzyme (data not shown). The assay was run in duplicate with BBM from each of the 80 hens. Samples without BBM addition were included as a control and to distinguish between inositol phosphate isomers (InsP_x_) produced by BBM from those contained in the feed or produced by plant-intrinsic phytases.

**TABLE 1:** Analyzed concentrations of inositol phosphates in the feed ingredients and the mixture before and after incubation in the *in vitro* assay without using BBM.

	Ins(1,2,5,6)P_4_	Ins(1,2,3,4,6)P_5_	Ins(1,2,3,4,5)P_5_	Ins(1,2,4,5,6)P_5_	InsP_6_
	*µ*mol/g				
Corn	n.d.^a^	n.d.	n.d.	0.3	8.9
Soybean meal	<LOQ^b^	0.3	0.8	1.4	15.8
Corn-soybean meal mixture	n.d.	n.d.	0.2	0.6	11.0
Incubation residue of corn-soybean meal mixture	n.d.	n.d.	0.4	0.7	11.3

^a^ n.d. = not detectable (< 0.1 *µ*mol/g).

^b^ <LOQ = below limit of quantification (0.3 *µ*mol/g).

Data are given as LSmeans; n = 4 for *in vitro* + InsP analysis of the controls.

The *in vitro* incubation was immediately followed by InsP_x_ extraction as described by Zeller et al. (2015) with slight modifications by Sommerfeld et al. (2018). The extracting agent containing 0.2 M EDTA and 0.2 M sodium fluoride (pH of 8; 4 °C) was added to the tube to a total volume of 1 mL. The pH value was adjusted to 8.0 by using NaOH (10%). The extraction was performed at 4 °C for 30 min under agitation (IKA KS 501 digital shakers with AS 501.1 Universal attachment, IKA-Werke GmbH & CO. KG, Staufen, Germany) and followed by centrifugation at 12,000 × *g* for 15 min (Thermo Scientific™ Heraeus Multifuge X3 FR, Fisher Scientific GmbH, Schwerte, Germany). The supernatant was collected in falcon tubes on ice. The pellet was re-suspended in 0.5 mL extracting agent and placed on ice on the shaker for another 30 min. After centrifugation at 12,000 × *g* for 15 min, supernatants from each sample were combined and 1 mL was pipetted into Eppendorf tubes. Samples were centrifuged at 14,000 × *g* for 15 min (Thermo Scientific™ Fresco™ 21, Fisher Scientific GmbH, Schwerte, Germany). The supernatant was filtered through a 0.2 *µ*m cellulose acetate filter (Chromafil® CA-20/25, Machery-Nagel, Düren, Germany) into a Microcon filter (cut-off 30 kDa) device (Amicon Ultra-0.5 Centrifugal Filter Unit, Millipore, Merck KGaA, Darmstadt, Germany) before the final centrifugation at 14,000 × *g* for 30 min. Measurement of InsP_3-6_ was done with high-performance ion chromatography (ICS-5000 system, Dionex, Thermo Scientific, Idstein, Germany). Separating enantiomers and distinguishing D- and L-forms was not possible with this method. The isomers Ins(1,2,6)P_3_, Ins(1,4,5)P_3_, and Ins(2,4,5)P_3_ could not be distinguished and are referred to as InsP_3x_ here. The detection of InsP_2_ isomers was done on the same analyzer but using other eluent concentrations. The InsP_6_ disappearance was calculated for each hen using the InsP_6_ concentrations in the corn-soybean meal mixture and the incubation residues.

### 2.4 Enzyme activity assay

Mucosal phosphatase activity was measured as described by Gonzalez-Uarquin et al. (2020) with modifications described by Novotny et al. (2023a). In brief, a mixture of distilled water, 25 *µ*g sodium phytate (6-0-123456-Na, Sirius fine chemicals SiChem GmbH, Bremen, Germany), and buffer (pH 5.5 from K-PHYT test kit [K-PHYT 05/19 assay; Megazyme International, Ireland]) was warmed to 40°C in a water bath (Shaking Water Baths GFL THERMOLAB 1070, GFL Gesellschaft für Labortechnik mbH, Burgwedel, Germany). After the addition of BBM solution equivalent to an amount of 160 *µ*g protein, the mixture was incubated for 15 min at 40 °C and pH 5.5. Then, the incubation was stopped with trichloric acid. Blind values were obtained in the same way but without the application of BBM and the stop reagent trichloric acid. Free inorganic phosphate (P_i_) was determined photometrically (Infinite® M Nano^+^, Tecan Group Ltd., Männdorf, Switzerland) at 655 nm and 40 °C as described in the method of the K-PHYT test kit. To calculate the P_i_ after BBM addition, values were corrected for the means of the blind values. Finally, measurements were standardized and expressed as *µ*mol P_i_ released by 1 g of BBM protein per min. The assay was run in duplicate with BBM from each of the 80 hens.

### 2.5 Statistical analysis

Data were analyzed with a 3-way ANOVA using the MIXED procedure of the software package SAS (version 9.4, SAS Institute Inc., Cary, North Carolina). The following model was used:

$$Y_{\text{ijklm}}=\mu +\alpha_{i}+\beta_{j}+\gamma_{k}+{\left(\alpha \beta \right)}_{\text{ij}}+{\left(\alpha \gamma \right)}_{\text{ik}}+{\left(\beta \gamma \right)}_{\text{jk}}+{\left(\alpha \beta \gamma \right)}_{\text{ijk}}+\delta_{l}+\phi_{m}+\varepsilon_{\text{ijklm}}$$

where Y_ijklm_ = response variable, μ = overall mean, α_i_ = effect of hen strain (fixed), β_j_ = effect of hen age (fixed), γ_k_ = effect of dietary P (fixed), all two- and three-way-interactions among hen strain, hen age, and dietary P (fixed), δ _l_ = block (random), ϕ_m_ = father/rooster (random), and ε_ijklm_ = residual error. The individual hen was the experimental unit. The results are presented as LSmeans and SEM. Correlations were calculated using the CORR procedure of SAS. Statistical significance was declared at *P* < 0.05. Results were visualized using GraphPad Prism (version 5.0, GraphPad Software Inc., San Diego, CA, United States).

## 3 Results

*In vitro* InsP_6_ disappearance during the incubation and concentrations of InsP isomers in the incubation residue were not affected by three- or two-way interactions of the factors dietary P, hen strain, and hen age (Table 2). The only factor exerting significant effects was hen age. The InsP_6_ disappearance in the *in vitro* assay was 1.0 *μ*mol/g lower when BBM of 19-week-old hens was used compared to 24-week-old hens (*P* < 0.001) (Table 3). The concentrations of InsP_6_, Ins(1,2,3,4,5)P_5_, Ins(1,2,3,4,6)P_5_, and Ins(1,2,3,4)P_4_ in the incubation residue were significantly higher when BBM of 19-week-old hens was used compared to BBM of 24-week-old hens (*P* ≤ 0.002). In contrast, InsP_3x_ and Ins(1,2)P_2_ concentrations in the incubation residue were significantly lower when BBM of 19-week-old hens was used (*P* ≤ 0.027) (Tables 2, 3). The coefficient of variation (CV%) of InsP_6_ concentration in the incubation residue ranged from 0% – 8% in samples incubated with BBM and 0% – 3% in samples incubated without BBM.

**TABLE 2:** Effects of hen age, dietary P, and hen strain on duodenal endogenous mucosal phosphatase activity and concentrations of inositol phosphates in the incubation residue and InsP_6_ disappearance in the three-step *in vitro* assay with BBM of laying hens (n = 10 hens per treatment). Main effects are shown in Table 3.

			Phosphatase activity	Ins(1,2)P_2_	InsP_3x_ ^1^	Ins(1,2,3,4)P_4_	Ins(1,2,5,6)P_4_	Ins(1,2,3,4,6)P_5_	Ins(1,2,3,4,5)P_5_	Ins(1,2,4,5,6)P_5_	InsP_6_	InsP_6_ disappearance
Hen age	Dietary P	Hen strain	*µ*mol P_i_/g BBM^2^ protein/min	*µ*mol/g								
19	P-^3^	LB^5^	2.8	1.1	1.8	1.7	0.2	0.3	0.6	0.1	6.5	4.5
19	P-	LSL^6^	3.2	1.1	1.7	1.7	0.2	0.3	0.6	0.2	6.6	4.4
19	P+^4^	LB	3.1	0.8	1.5	1.7	0.2	0.3	0.7	0.2	6.9	4.1
19	P+	LSL	3.4	0.9	1.7	1.8	0.2	0.3	0.7	<LOQ	6.8	4.3
24	P-	LB	3.7	2.2	1.9	1.3	<LOQ^7^	0.2	0.4	n.d.^8^	5.7	5.3
24	P-	LSL	3.7	1.9	1.9	1.3	<LOQ	<LOQ	0.4	n.d.	5.9	5.1
24	P+	LB	4.4	2.8	1.9	1.2	<LOQ	0.2	0.3	n.d.	5.5	5.5
24	P+	LSL	3.8	2.1	1.8	1.3	0.2	0.2	0.5	n.d.	5.9	5.1
		SEM	0.37	0.31	0.14	0.11	0.02	0.03	0.06	0.02	0.27	0.27
*P* values	Age		0.002	<0.001	0.027	<0.001	0.055	0.002	<0.001	.	<0.001	<0.001
	Strain		0.921	0.325	0.920	0.549	1.000	0.807	0.434	0.370	0.494	0.494
	P		0.246	0.856	0.334	0.947	0.488	0.852	0.666	0.190	0.645	0.645
	Strain × P		0.426	0.699	0.787	0.552	1.000	0.430	0.666	.	0.967	0.967
	Age × P		0.852	0.133	0.871	0.391	.	0.171	0.357	.	0.323	0.323
	Age × strain		0.183	0.207	0.554	1.000	.	0.325	0.297	.	0.494	0.494
	Age × Strain × P		0.667	0.586	0.334	0.947	.	.	0.297	.	0.494	0.494

1 Ins(1,2,6)P_3_, Ins(1,4,5)P_3_, and Ins(2,4,5)P_3_ could not be differentiated due to co-elution and are thus referred to as InsP_3x_.

2 BBM = brush border membrane.

3 P- = without mineral P supplement.

4 P+ = with 1 g supplement P/kg.

5 LB = Lohmann Brown-classic.

6 LSL = Lohmann LSL-classic.

7 <LOQ = below limit of quantification (for Ins(1,2,5,6)P_4_ and Ins(1,2,3,4,6)P_5_ 0.3 *µ*mol/g and for Ins(1,2,4,5,6)P_5_ 0.2 *µ*mol/g).

8 n.d. = not detectable (< 0.1 *µ*mol/g).

Data are given as LSmeans.

**TABLE 3:** Main effects of hen age, dietary P, and hen strain on duodenal endogenous mucosal phosphatase activity and concentrations of inositol phosphates in the incubation residue and InsP_6_ disappearance in the three-step *in vitro* assay with BBM of laying hens (n = 40 hens per factor, 80 in total).

			Phosphatase activity	Ins(1,2)P_2_	InsP_3x_ ^1^	Ins(1,2,3,4)P_4_	Ins(1,2,5,6)P_4_	Ins(1,2,3,4,6)P_5_	Ins(1,2,3,4,5)P_5_	Ins(1,2,4,5,6)P_5_	InsP_6_	InsP_6_ disappearance
Hen age	Dietary P	Hen strain	*µ*mol P_i_/g BBM^2^ protein/min	*µ*mol/g								
19			3.1^a^	1.0^a^	1.7^a^	1.7^a^	0.2	0.3^a^	0.7^a^	<LOQ	6.7^a^	4.3^a^
24			3.9^b^	2.2^b^	1.9^b^	1.3^b^	<LOQ^7^	<LOQ^b^	0.4^b^	n.d.^8^	5.7^b^	5.3^b^
SEM			0.21	0.16	0.08	0.06	0.01	0.02	0.04	.	0.15	0.15
	P-^3^		3.4	1.6	1.8	1.5	<LOQ	<LOQ	0.5	0.2	6.2	4.8
	P+^4^		3.6	1.6	1.7	1.5	<LOQ	0.2	0.5	n.d.	6.3	4.7
	SEM		0.21	0.16	0.08	0.06	.	0.02	0.04	0.01	0.15	0.15
		LB^5^	3.5	1.7	1.8	1.5	<LOQ	0.2	0.5	0.2	6.2	4.8
		LSL^6^	3.5	1.5	1.8	1.5	<LOQ	<LOQ	0.6	n.d.	6.3	4.7
		SEM	0.21	0.16	0.08	0.06	.	0.02	0.04	0.01	0.15	0.15

^a^ ^,b^Different superscript lowercase letters within a column indicate significant effects of age.

1 Ins(1,2,6)P_3_, Ins(1,4,5)P_3_, and Ins(2,4,5)P_3_ could not be differentiated due to co-elution and are thus referred to as InsP_3x_.

2 BBM = brush border membrane.

3 P- = without mineral P supplement.

4 P+ = with 1 g supplement P/kg.

5 LB = Lohmann Brown-classic.

6 LSL = Lohmann LSL-classic.

7 LOQ = limit of quantification (for Ins(1,2,5,6)P_4_ and Ins(1,2,3,4,6)P_5_ 0.3 *µ*mol/g and for Ins(1,2,4,5,6)P_5_ 0.2 *µ*mol/g).

8 n.d. = not detectable (< 0.1 *µ*mol/g).

Data are given as LSmeans.

The mucosal phosphatase activity of the BBM was only affected by the hen age (*P* < 0.002) and no three- or two-way interactions of the factors dietary P, hen strain, and hen age were found (Table 2). The 24-week-old hens had, on average, 0.8 *µ*mol P_i_/g BBM protein/min higher phosphatase activity than the 19-week-old hens (Table 3).

## 4 Discussion

This study aimed to characterize the endogenous mucosal phosphatases of laying hens by assessing their enzymatic activity and degradation products. Currently, it remains unclear whether endogenous mucosal phosphatases exhibit exclusively phytase activity or also possess broader phosphatase activity. While established degradation pathways describe the breakdown of phytate by phytases (Greiner et al., 1993), the reduction in lower InsP isomers in the present study could result either from phytases with additional phosphatase activity or from the action of other phosphatases, such as alkaline phosphatase located in the BBM.

For the first time, enzyme activity measurements were combined with a complementary *in vitro* assay to measure the InsP_6_ degradation products, using the same BBM preparations in both assays. While enriched BBM characterize cell-wall associated activities that may not fully reflect enzyme activity in the lumen, this approach attempted to address the challenge of *in vivo* enzyme characterization. Here, enzymes from various origins (microbial, plant, and mucosal) coexist in the digestive tract, posing a challenge in the identification of the specific contribution of mucosal enzymes to InsP_6_ degradation. Notably, such differentiation is only possible in germ-free birds and at complete inactivation of plant intrinsic enzymes. Of note, the two assays are unable to replicate the mucosal microclimate as it occurs *in vivo* which develops beneath the mucosa layer (Akiba et al., 2007). Its function is to protect epithelial cells and adherent enzymes (Akiba et al., 2007) from fluctuating pH in digesta (Chikina and Matic Vignjevic, 2021). According to Rechkemmer et al. (1986), the pH in this microclimatic environment was in the neutral range in guinea pigs and rats, but for poultry, it has not been investigated yet. To explore variation, BBM was isolated from hens of varying ages undergoing different dietary treatments. The findings showed that the age of the hens influenced the results, whereas the genetic strain and the dietary mineral P supplementation did not.

### 4.1 Evaluation of the *in vitro* InsP_6_ degradation assay

Phosphatase activity assays are commonly carried out using sodium phytate as a substrate and at one distinct pH. However, the three-step *in vitro* InsP_6_ degradation assay used in the present study employed common feed ingredients as phytate matrix and applied various pH values to simulate the digesta in different segments of the gastrointestinal tract. By introducing BBM from laying hens in the third step (simulation of the small intestine), the mucosal phosphatases can exert their effect without interfering with gut microbial phosphatases. To test the sole presence of mucosal phosphatases in the assay, proteomic analysis of a BBM sample confirmed the absence of microbial phosphatases (data not shown).

The reproducibility of the modified *in vitro* assay was supported by CV% values. Values below 8% for InsP_6_ concentrations in the incubation residue indicated robust reproducibility when using BBM-derived mucosal phosphatases. These findings align with the CV% results reported by Sommerfeld et al. (2017) for the original, unmodified *in vitro* assay, further substantiating the reliability of this approach.

**FIGURE 1:**
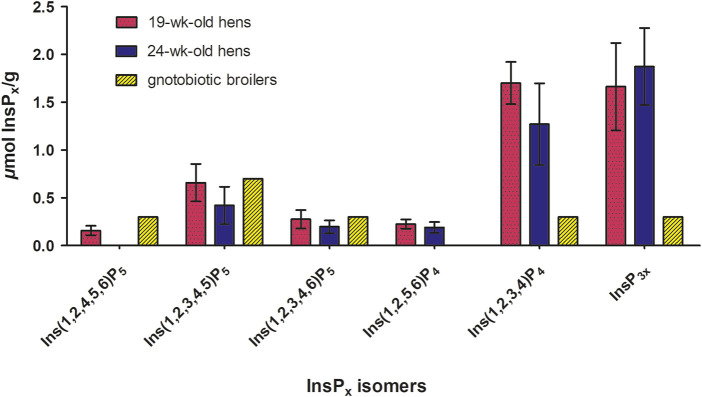
Concentrations of inositol phosphate isomers (InsP_5_-InsP_3_) (*µ*mol/g fresh matter) in the incubation residue after *in vitro* incubation with BBM for both hen ages (n = 40 hens per age, LSmeans ± SD) (Table 3) and concentrations of inositol phosphate isomers (InsP_5_-InsP_3_) (*µ*mol/g fresh matter) in the duodenum + jejunum chyme of gnotobiotic broilers fed without phytase and mineral P and with a reduced Ca level conducted by Sommerfeld et al. (2019) (n = 10 birds, treatment means). The broilers were hatched germ-free in isolators, ensuring the absence of microbiota. Thus, the phytate degradation in the germ-free animals can solely be ascribed to endogenous mucosal enzymes.

The InsP_x_ isomer patterns determined in the incubation residue after *in vitro* incubation with BBM from laying hens agreed with those reported in studies with gnotobiotic broiler chickens (Sommerfeld et al., 2019). To facilitate the comparison of the InsP isomers detected after *in vitro* incubation, data from the gnotobiotic broiler study conducted by Sommerfeld et al. (2019) were incorporated and are presented together (Figure 1). Broilers were hatched and raised in germ-free isolators ensuring the absence of microbiota in the intestine of these broilers. This study by Sommerfeld et al. (2019) serves for comparison, as the phytate degradation in the germ-free animals can solely be ascribed to endogenous mucosal enzymes. Most degradation products found in the incubation residue qualitatively matched those found in the duodenal and jejunal digesta of gnotobiotic broilers fed diets without phytase, without mineral P, and with a reduced Ca level (Figure 1), confirming generally the InsP isomers produced by mucosal phosphatases of both laying hens and broilers. Both the gnotobiotic broiler trial by Sommerfeld et al. (2019) and the modified *in vitro* assay were conducted in the absence of microorganisms, enabling focused insights into the characteristics of endogenous mucosal phosphatases. The quantitative amount of Ins(1,2,3,4)P_4_ and InsP_3x_, however, differed between the *in vitro* results and *in vivo* results of the gnotobiotic broilers. The conditions in the *in vitro* assay are still considerably different from those *in vivo*. The closed *in vitro* system without absorption processes cannot completely reproduce the dynamic processes in the small intestine. Further, the phytate matrix differed, being a corn-soybean meal mixture *in vitro* and a completely formulated diet with Ca (and P) supplements *in vivo*, which could have led to different results. Furthermore, Siegert et al. (2023) observed the same InsP_6-4_ isomers in the excreta of cecectomized laying hens fed a diet devoid of mineral P and phytase as in the present study, but again only qualitatively. The ceca of poultry are characterized by high microbial InsP_6_ degradation (Zeller et al., 2015). Although some gut microbial activity may contribute to InsP_6_ hydrolysis in cecectomized laying hens, the results of Siegert et al. (2023) support the existence of mucosal enzyme activity in laying hens. The modified *in vitro* assay using BBM offers the possibility of testing a broad range of materials *in vitro*, which might not be feasible with gnotobiotic animals *in vivo*. It is a suitable tool for investigating mucosal phosphatase activity under controlled conditions, with the awareness that the *in vivo* conditions, including the mucosa microenvironment, cannot be simulated in its entirety.

### 4.2 Degradation pathway of endogenous mucosal phosphatases

Phytases initiate the dephosphorylation of phytate at specific positions on the inositol ring, and the starting position determines their classifications (e.g., 3-phytase or 6-phytase). Not only the starting point of dephosphorylation is characteristic, but also the InsP degradation pattern is unique and predetermined for each phosphatase (Greiner et al., 1993). Using BBM in the three-step *in vitro* assay, the degradation products of the mucosal enzymes (phytase and other phosphatases) were identified, enabling their characterization and revealing potential variations among hens of different age, strain, and diets. To distinguish between the effect of the mucosal phosphatases and those from the feed, control incubations were conducted without the addition of BBM. In these controls, the corn-soybean meal mixture contained Ins(1,2,4,5,6)P_5_ and Ins(1,2,3,4,5)P_5_ both before and after incubation with no further degradation observed, indicating negligible plant phytase activity, which was expected from the use of corn and soybean meal. In contrast, when using BBM in the assay, Ins(1,2,4,5,6)P_5_ was present only in trace amounts in the incubation residue, suggesting further dephosphorylation occurred. Notably, the addition of BBM resulted in the formation of Ins(1,2,3,4,5)P_5_ and Ins(1,2,3,4,6)P_5_, whereas Ins(1,2,3,4,6)P_5_ was not present in the feed ingredients. These findings are consistent with previous studies reporting these two InsP_5_ isomers to appear in the ileal digesta of broiler chickens fed diets without exogenous phytase, as summarized by Rodehutscord et al. (2022). This similarity supports the view that the same types of endogenous phosphatases are active in broiler chickens and laying hens.

**FIGURE 2:**
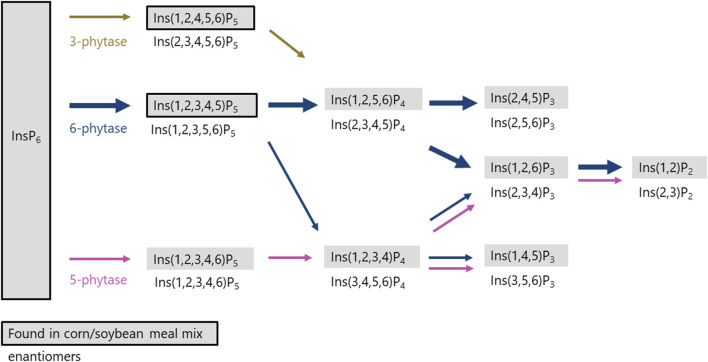
Proposed *in vitro* degradation pathway (InsP_6_ to InsP_2_) in the incubation residue after three-step *in vitro* incubation with BBM of laying hens. Isomers in grey boxes were detected by analysis in incubation residue with BBM (Table 3). Isomers in grey boxes with black frames were also found in incubation residue of controls without BBM (Table 1). Isomers underneath boxes are the appropriate enantiomers of the detected isomers.

The appearance of Ins(1,2,3,4,5)P_5_ in the incubation residue solely after incubation with BBM indicated the activity of a 6-phytase (Figure 2). Greiner et al. (2001) described the dephosphorylation pathway of the microbial 6-phytase of *Escherichia coli* with Ins(1,2,3,4,5)P_5_ as the first dephosphorylation product followed by Ins(2,3,4,5)P_4_. The detection of Ins(1,2,5,6)P_4_, the enantiomer of Ins(2,3,4,5)P_4_, in the incubation residue might therefore be unexpected. However, studies by our working group using 6-phytases in diets for broilers (Sommerfeld et al., 2018; Krieg et al., 2020) and laying hens (Siegert et al., 2023) also observed an accumulation of Ins(1,2,5,6)P_4_ in intestinal digesta or excreta. As Ins(1,2,5,6)P_4_ is used as a standard and the analytical method does not allow separation of enantiomers, the detected Ins(1,2,5,6)P_4_ likely corresponds to its enantiomer Ins(2,3,4,5)P_4_ and is thus a 6-phytase product (Zeller et al., 2015). Alternatively, Ins(1,2,5,6)P_4_ could also have been a dephosphorylation product of Ins(1,2,4,5,6)P_5_ derived from the feed ingredients, dephosphorylated by a 3-phytase (Greiner and Konietzny, 2010) (Figure 2). As previously shown by Konietzny and Greiner (2002) for microbial 6-phytases, the subsequent dephosphorylation to InsP_3_ isomers might imply the formation of Ins(2,4,5)P_3_ and Ins(1,2,6)P_3_ or their enantiomers. While InsP_3_ isomers are not clearly distinguishable with the analytical method used, the isomers Ins(1,2,6)P_3_, Ins(1,4,5)P_3_, and Ins(2,4,5)P_3_ may have occurred in the incubation residue without precise quantification of their distribution and reliable occurrence, at least one of the isomers was detected. Likewise, the isomer Ins(2,4,5)P_3_ detected by Greiner et al. (2001) and the isomers Ins(1,2,6)P_3_ and Ins(2,4,5)P_3_ described by Konietzny and Greiner (2002) were included in the InsP_3_ mixture. Among the InsP_2_ isomers, only Ins(1,2)P_2_ was detected after incubation with BBM of which the enantiomer might again be the product of a 6-phytase (Greiner et al., 2001; Greiner and Konietzny, 2010). Another possible minor pathway of a 6-phytase involves the dephosphorylation of (1,2,3,4,5)P_5_, producing Ins(1,2,3,4)P_4_ as an intermediate (Figure 2). Zeller et al. (2015) described Ins(1,2,3,4)P_4_ as a minor degradation product of 6-phytases. Further dephosphorylation of Ins(1,2,3,4)P_4_ in the incubation residue could yield Ins(1,2,6)P_3_ or Ins(1,4,5)P_3_. Along this line, the degradation of InsP by 6-phytase appears to produce Ins(1,2)P_2_ or rather its enantiomer Ins(2,3)P_2_.

The detection of Ins(1,2,3,4,6)P_5_ in the incubation residue, which was absent in the feed ingredients, indicates the activity of a 5-phytase (Figure 2). The activity of a 5-phytase in poultry has been previously shown in a broiler study (Sommerfeld et al., 2019). Furthermore, Rodehutscord et al. (2022), summarizing several broiler studies, reported the occurrence of Ins(1,2,3,4,6)P_5_ in ileal digesta even when it was not present in the feed. The isomer Ins(1,2,3,4)P_4_, detected in the present study after *in vitro* incubation with BBM, aligns with a degradation pathway previously described by Konietzny and Greiner (2002) for 5-phytase in lily pollen. A similar pathway was also reported as a minor degradation route in wheat by Sun et al. (2017). Subsequent dephosphorylation along this pathway could lead to Ins(1,4,5)P_3_ or Ins(1,2)P_2_, or their enantiomers, consistent with degradation mediated by 5-phytase and other phosphatases.

Considering all observed dephosphorylation products during *in vitro* incubation with BBM and the measured concentrations of InsP_5_ isomers, the results suggest that the active endogenous mucosal enzymes can be classified as primarily 6- and secondarily 5-phytases.

### 4.3 Effects of laying hen strain, hen age, and dietary P

No effects of laying hen strain and dietary P level on phosphatase activity and InsP degradation were found. However, hen age significantly influenced the results of both *in vitro* assays. In 19-week-old hens, BBM phosphatase activity and InsP_6_ disappearance in the incubation residue were significantly lower compared to the 24-week-old hens (Table 3). This age-related difference is likely attributable to physiological changes occurring between 19 and 24 weeks of age. During this period, hens’ transition from growth only to growth and egg production leads to an associated change in nutrient requirements, which may be a decisive factor in this context. The impact of this transition on phosphatase activity has been sparsely studied. Marounek et al. (2008) reported a numerically lower phytase activity in 20-week-old laying hens compared to 47-week-old laying hens, though the activity was measured in the whole mucosa rather than enriched BBM. Other studies have also reported age-related effects, whereby the younger hens were typically 24 weeks old or older (Abudabos, 2012; Sommerfeld et al., 2020) and already at full egg production.

**FIGURE 3:**
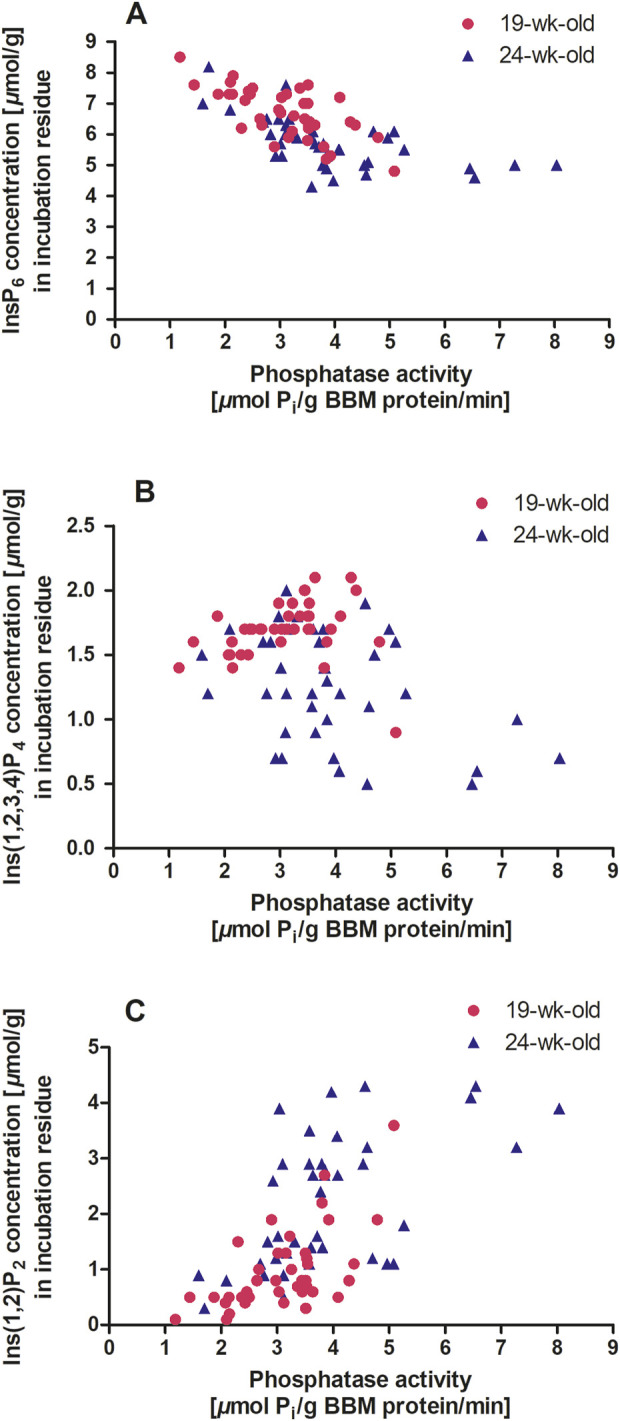
**(A–C)** Duodenum BBM phosphatase activity (*µ*mol P_i_/g BBM protein/min) and inositol phosphate concentration in the incubation residue (*µ*mol/g) after *in vitro* incubation with BBM. The symbols indicate data points of individual hens (n = 40 hens per age, 80 in total; GraphPad Prism 5.0). A, 19 weeks old hens: y = - 0.65 x + 8.71, *r* ^2^ = 0.47, *P* < 0.001 and 24 weeks old hens: y = - 0.37x + 7.17, *r* ^2^ = 0.36, *P* < 0.001. B, 19 weeks old hens: y = 0.04 x + 1.56, *r* ^2^ = 0.03, *P* = 0.285 and 24 weeks old hens: y = - 0.12 x + 1.75, *r* ^2^ = 0.16, *P* = 0.012. C, 19 weeks old hens: y = 0.52 x – 0.63, *r* ^2^ = 0.38, *P* < 0.001 and 24 weeks old hens y = 0.49 x + 0.30, *r* ^2^ = 0.32, *P* < 0.001.

When the results of the two assays were combined, higher phosphatase activity was associated with a lower InsP_6_ concentration in the incubation residue for both 19-week-old hens (r = −0.689; *P* < 0.001) and 24-week-old hens (r = −0.604; *P* < 0.001) (Figure 3A). However, in four hens that exerted a phosphatase activity of more than 6 *µ*mol P_i_/g BBM protein/min, the InsP_6_ concentration was not further reduced. Perhaps the P_i_ release inhibited further InsP_6_ dephosphorylation (Angel et al., 2002), or the remaining InsP_6_ was less accessible for the enzyme. However, this should be discussed cautiously because of the low number of observations in that activity range.

The relationship between lower InsP isomers and phosphatase activity differed markedly between the two age groups. In 19-week-old hens, Ins(1,2,3,4)P_4_ appeared to be independent of phosphatase activity (Figure 3B). In contrast, 24-week-old hens showed a negative correlation between phosphatase activity and Ins(1,2,3,4)P_4_ concentration in the incubation residue (r = −0.394; *P* = 0.012). For InsP_3x_, a highly positive correlation with phosphatase activity was observed in 19-week-old hens (r = 0.628; *P* < 0.001) but not in 24-week-old hens. Bedford and Rousseau (2017) identified InsP_4_ and InsP_3_ as bottlenecks in further degradation to *myo*-inositol when using phytases. These bottlenecks could only be bypassed with high amounts of phytase, as endogenous phosphatases could not perform the necessary degradation. In the present study, conducted without phytase supplementation, the bottleneck at InsP_4_ and InsP_3_ that was seen in 19-week-old hens, was not present in 24-week-old hens, but a higher InsP_2_ concentration suggests a shift to lower phosphorylated InsP due to increased phosphatase activity at this age (Table 3). This relationship was reflected in the correlation between phosphatase activity and Ins(1,2)P_2_ concentration in the incubation residue of the *in vitro* incubation for both 19-week-old hens (r = 0.615; *P* < 0.001) and 24-week-old hens (r = 0.565; *P* < 0.001) (Figure 3C).

Applegate et al. (2003) described an inverse relationship between small intestine brush border vesicle phytase activity and dietary Ca concentration in poultry. The Ca concentration in intestinal digesta of the hens used in the present study was studied by Sommerfeld et al. (2024). Negative correlations between phosphatase activity of the 19-week-old hens and Ca concentration in the digesta of duodenum + jejunum (r = −0.409; *P* < 0.009) and ileum (r = −0.418; *P* < 0.009) were found, similar to the results of Applegate et al. (2003). However, no such correlations were found for 24-week-old hens. None of the 19-week-old hens were laying eggs, resulting in a lower Ca requirement than in 24-week-old hens. Because the same diets were used at both ages, the Ca concentration was consistently higher in the digesta of the duodenum + jejunum in wk 19 than week 24 (Sommerfeld et al., 2024). The mucosal phosphatase activity can be influenced by dietary Ca and P concentrations (Novotny et al., 2023b; Davies et al., 1970). Brun et al. (2006) demonstrated that increasing Ca concentration in rat diets led to the binding of Ca to intestinal alkaline phosphatase and reduced its activity. Moreover, excessive Ca might have reduced the structure stability of intestinal alkaline phosphatase (Brun et al., 2009). This might explain the reduced phosphatase activity in 19-week-old hens, as Ca binding to the phosphatases likely reduced their activity. Another indirect effect of Ca on phosphatase activity could stem from the formation of InsP_6_-Ca complexes (Angel et al., 2002; Tamim et al., 2004; Selle et al., 2009; Krieg et al., 2021). High dietary Ca concentrations and consequently increased concentrations in the digesta promote the formation of these InsP_6_-Ca complexes (Angel et al., 2002), which are less soluble (Applegate et al., 2003). Reduced solubility might limit substrate availability for phosphatases, thereby diminishing their activity (Angel et al., 2002). This was also shown by Van der Klis et al. (1997) for 24-week-old laying hens where higher Ca levels of the diet (40 g Ca/kg) compared to lower concentrations (30 g Ca/kg) reduced the ileal phytate degradation. In 24-week-old hens, phosphatase activity was negatively correlated with the measured P concentration in the digesta of the duodenum + jejunum (r = −0.342; *P* < 0.031). Consistently Onyango et al. (2006) reported a negative effect of dietary P (from dicalcium phosphate) on the activity of intestinal mucosal phytase. However, no such correlation was found in 19-week-old hens in the present study. Although P can potentially inhibit endogenous phosphatase activity via end-product inhibition, this effect was not evident in the present study. The dietary P level likewise had no impact on the phosphatase activity and InsP degradation. This was in contrast to the findings by Davies et al. (1970) who showed a depressing effect of added P on the intestinal phytase activity; however, they also mentioned an increased amount of Ca in the diet has a possible decreasing effect on the intestinal phosphatase activity, matching the previously mentioned correlations with Ca for the 19-week-old hens. Possibly the intestinal digesta Ca concentrations overlaid the effect of the dietary P.

Contrary to expectations due to higher activities in LB or Hy-line Brown hens than in LSL or Hy-line White W-36 in the jejunum or duodenum samples, as described by Sommerfeld et al. (2020) and Abudabos (2012), the different genetic strains had no effect on the phosphatase activity in the present study. The activity was numerically but not significantly higher for the LB hens compared to the LSL hens at both ages. Presumably, the laying hen age had a greater influence on the activity and layered the effect of the strain.

## 5 Conclusion

The InsP_x_ patterns in the incubation residue after the three-step *in vitro* incubation indicated the activity primarily of a 6-phytase and secondarily of a 5-phytase in the duodenal mucosa. The consistent results from both *in vitro* assays–higher phosphatase activity and higher InsP degradation in 24-week-old hens–provide a comprehensive characterization of these enzymes. Under the conditions of this study, the mucosal enzyme activity was not affected by the addition of mineral P to the feed. However, higher Ca concentration in the small intestine may have been relevant, especially in 19-week-old hens. Further studies on the influence of Ca on mucosal phosphatases *in vitro* may contribute to clarifying the differences between the ages. The metabolic changes associated with the sexual maturation of the hens and related differences in nutrient metabolism warrant further investigation.

## Data Availability

The original contributions presented in the study are included in the article/supplementary material, further inquiries can be directed to the corresponding authors.
